# Tracing Key Molecular Regulators of Lipid Biosynthesis in Tuber Development of *Cyperus esculentus* Using Transcriptomics and Lipidomics Profiling

**DOI:** 10.3390/genes12101492

**Published:** 2021-09-24

**Authors:** Liyan Wang, Meiling Jing, Naveed Ahmad, Yifei Wang, Yijin Wang, Jia Li, Xiaowei Li, Weican Liu, Nan Wang, Fawei Wang, Yuanyuan Dong, Haiyan Li

**Affiliations:** 1 Engineering Research Center of Bioreactor and Pharmaceutical Development, College of Life Sciences, Jilin Agricultural University, Changchun 130118, China; wly19940427sci@163.com (L.W.); 15144363778@163.com (M.J.); naveed@jlau.edu.cn (N.A.); wangyifei1104@outlook.com (Y.W.); 15506007439@163.com (Y.W.); L15043193095@163.com (J.L.); xiaoweili1206@jlau.edu.cn (X.L.); weicanliu@jlau.edu.cn (W.L.); wangnanlunwen@jlau.edu.cn (N.W.); wangfawei@jlau.edu.cn (F.W.); 2 Institute of Crop Germplasm Resources, Shandong Academy of Agricultural Sciences, Jinan 250100, China; 3 College of Tropical Crops, Hainan University, Haikou 570228, China

**Keywords:** *Cyperus esculentus*, lipidomics, lipid synthesis, triacylglycerol, RNA-seq, *Saccharomyces cerevisiae*, *CePAH1*

## Abstract

*Cyperus esculentus* is widely representing one of the important oil crops around the world, which provides valuable resources of edible tubers called tiger nut. The chemical composition and high ability to produce fats emphasize the role of tiger nut in promoting oil crop productivity. However, the underlying molecular mechanism of the production and accumulation of lipids in tiger nut development still remains unclear. Here, we conducted comprehensive transcriptomics and lipidomics analyses at different developmental stages of tuber in *Cyperus esculentus*. Lipidomic analyses confirmed that the accumulation of lipids including glycolipids, phospholipids, and glycerides were significantly enriched during tuber development from early to mature stage. The proportion of phosphatidylcholines (PC) declined during all stages and phosphatidyl ethanolamine (PE) was significantly declined in early and middle stages. These findings implied that PC is actively involved in triacylglycerol (TAG) biosynthesis during the tubers development, whereas PE may participate in TAG metabolism during early and middle stages. Comparative transcriptomics analyses indicated several genomic and metabolic pathways associated with lipid metabolism during tuber development in tiger nut. The Pearson correlation analysis showed that TAG synthesis in different developmental stages was attributed to 37 candidate transcripts including *CePAH1*. The up-regulation of diacylglycerol (DAG) and oil content in yeast, resulted from the inducible expression of exogenous *CePAH1* confirmed the central role of this candidate gene in lipid metabolism. Our results demonstrated the foundation of an integrative metabolic model for understanding the molecular mechanism of tuber development in tiger nut, in which lipid biosynthesis plays a central role.

## 1. Introduction

Oil plants constitute a key source of nutrients and energy for humans and animals. Due to the limited cultivated land, and the increasing demand for oil consumption, novel oil plants or genetic breeding varieties with high oil content, short growing season, and wide adaptability have been receiving increasing attention as an alternative resource to traditional oil crops. *Cyperus esculentus* also known as tiger nut is a monocotyledonous perennial plant and a member of the family of *cyperaceae*. The morphology of tiger nut is similar to that of the grasses or rushes appearance, but it usually exhibits a hypogeal rhizome similar to that of potato. Tiger nut is a prominent source of lipids, oleic acid, carbohydrates, vitamin E, β-carotene, and mineral substances with high crude oil content, which is about 24.9% to 28.9% [1]. Tiger nut is also an easy and fast-growing plant, with approximately 110 days cultivated period, presenting a high average yield ranged from 4.5 to 12 t ha^−1^ depending on the ambient temperature. Due to its high oil content, high nutritional value, and perfect animal feed, the tiger nut is emerging as a novel economic oil plant with expanded worldwide cultivation including Africa, Australia, China, and Spain. As a valuable resource of lipids, the tiger nut remains underestimated and its potential in the aspect of lipids production and accumulation needs further investigations.

The first electrospray ionization mass spectrometry (ESI-MS) on lipidomics reported various phospholipid molecules through quantitative analysis. The study importantly revealed the physiological importance of lipids [2], identified their associated metabolic networks, and further demonstrated their involvement in multiple signaling pathways of eukaryotic cells. Over the last decades, ESI-MS usage has experienced considerable improvements in plant biotechnology, accelerating the research rhythmic on lipidomic. Similar techniques have been applied to descript the regulation of lipid modeling to various plant tissues and such studies have been conducted on several plants [3]. Lipidomic was also used to restore lipid modeling species under varied growth conditions, providing insights into the biochemical features of lipid metabolism during plant tissue development. 

The modulation and accumulation of lipids plays a very important role in plant tissue development. The lipidomic analysis identified existing correlations among varied polar lipids including phosphatidylcholines (PC), phosphatidyl ethanolamine (PE), and some neutral lipids such as diacylglycerol (DAG) and triacylglycerols (TAG). In addition, lipidomic researches were also implemented in the successful characterization of certain groups of unsaturated lipids [4,5]. In previous studies, metabolic pathways regulating fatty acids synthesis and lipids metabolism in oilseed plants such as *Arabidopsis thalinia* (L.) and *Brassica napus* (L.) were achieved through the application of enzymology coordinated with the flux of carbon [6]. The general mechanism mediating the lipids production was containing high levels of lipid molecules such as PC, DAG, or TAG, which are the most prominent precursors of lipids in plants. However, the signaling pathway involved in TAG synthesis through the Kennedy pathway appears to be distinct in terms of the flux of fatty acids and enzymology among plant species [7].

Transcriptional analyses in many plant models have shown that the regulation of seed oil production depends on the developmental stages [8]. Many enzymes and transcriptional factors involved in TAG biosynthetic pathways were identified and confirmed as positive regulators in various plant species. Among those, fatty acids synthesis (PUSH), *FAD2*, *FAD3* [9], *KCSs* [10], or *MYB1* [11] represent the key regulators of fatty acids composition alteration. For instance, *FAX1* and *FAX2* facilitate fatty acids transport from plastids to improve seed oil accumulation [12,13]. In the Kennedy pathway associated with TAG and membrane lipid synthesis (PULL), acyltransferases such as Lysophosphatidic acid acyltransferase (LPAAT) [14], diglyceride acyltransferase (DGAT) [15], and Phospholipid diacylglycerol acyltransferase1 (PDAT1) [16] are important for TAG biosynthesis. Recent lipid accumulation studies revealed that DGAT1 and DGAT2 in *Cyperus esculentus* strongly affected TAG production and lipid accumulation [17]. The *SDP1* also regulates TAG accumulation by maintaining FA turnover in *Arabidopsis* *thalinia* [18]. Several transcription factors likewise *APETALA2* transcription factor family, *WRI1* [19], *MYB96* [20], B3-type contained DNA binding domain *FUSCA3* [21], and *Lec1* [22], were all reported as the key regulators initiating lipid assembly in higher plants.

Although several studies focused on understanding lipid remodeling and signal transduction pathways in model plants related to plant growth and development, there is limited information available on the regulatory mechanism governing the production, regulation, and accumulation of lipids congruent with transcriptomic analyses. Thus, it is fundamental to investigate different molecular pathways that regulate various aspects of lipid metabolism and further facilitate the genetic improvement of the oil plants production. In this study, we conducted comparative lipidomics and transcriptomics analyses at different developmental stages of tubers in tiger nut, and then explored candidate gene expression profiles during lipid metabolism. The understanding of the alternations between lipid categories and biological mechanisms of oil biosynthesis in tiger nut tubers may provides a landscape of gene-metabolic network on deciphering the underlying molecular regulatory mechanism of lipid metabolism and plant growth and development. In addition, it also facilitates tiger nut germplasm utilization and other plant breeding programs. 

## 2. Materials and Methods

### 2.1. Plant Materials and Treatments

The sampled plants of *Cyperus esculentus* were cultivated in Jilin province, China, under natural conditions on 14 May 2019. Based on observations on the *Cyperus esculentus* tuber’s development, five developmental stages were classified in this study. We namely arranged these five developmental stages as the 35 days after sowing (initial stage, 35 DAS); 50 days after sowing (early stage, 50 DAS); 70 days after sowing (middle stage, 70 DAS); 90 days after sowing (late stage, 90 DAS) and finally, 120 days after sowing (mature stage, 120 DAS). Comparative transcriptome analysis was further conducted to explore gene expression patterns during lipid biosynthesis in the tubers of *Cyperus esculentus.* For this step, samples (tubers) were collected in three replicates for each developmental stage (five developmental stages), then immediately froze the collected materials in liquid nitrogen and stored at −80 °C until further uses. 

### 2.2. Measurement of Oil Content

Five milligrams (5 mg) of dry seeds were used for oil content and fatty acid analysis. After placing all seeds into a glass tube, we added 1 mL of 2.5% sulfuric acid-methanol solution, 0.4 mL of toluene, and 0.2 mL of 2 mg/mL C17:0 solutions in toluene. The final solution was therefore mixed by vortex and heated at 90 °C water bath for 1 h; then, 1.8 mL of ddH_2_O and 1 mL of hexane were added after the tube was cooled down. After 15 min, the supernatant was filtered using a 0.45 μm microporous membrane. The filtrate was used to determine the fatty acid GC (Gas Chromatography) content using an Agilent 7890A instrument (Agilent Technologies, Inc., Wilmington, DE, USA). At least three replicate samples were examined for all of the experiments.

### 2.3. Nile Red Staining

Five development tubers were collected, peeled, cut into small pieces of 0.5 × 0.5 cm, and then transferred into a fix solution of 4% tissues for 24 h at 4 °C. Then, the tissues were embedded into paraffin by use embedding cassettes, blocked, put at 4 °C until complete solidification, and cut at 5–10 μm, dewaxing with Electro Thermostatic Blast Oven (Henan Lanphan Technology Co. Ltd., Zhengzhou, China) at 60 °C. The sections were deparaffinized, rehydrated, and stained with Nile Red (Invitrogen, Waltham, MA, USA) for a period of 2 min. Sections were then rinsed in running tap water, dehydrated, mounted, and captured, the sheet was sealed with neutral gum at the end.

### 2.4. Lipid Extraction

For the lipid extraction, 100 mg of each sample were transferred into the 5 mL centrifuge tubes, in which we added 1500 μL of mixed solution of chloroform and methanol (2:1) pre-cooled at −20 °C and divided in five steel balls (all insufficient samples size were reduced to an equal scale). Furthermore, we grinded all samples using a high flux organization grinding apparatus for 1.5 min at 60 Hz, put on the ice for 30 min, add 0.38 mL ddH_2_O, vortex for 30 s, and put back on the ice for 10 min, centrifuged at 12,000 rpm for 5 min at room temperature and transfer 0.6 mL lower layer fluid into a new centrifuge tube, add 1000 μL of previous mixed solution, vortex for 30 s, centrifuged at 12,000 rpm for 5 min at room temperature and transfer 0.8 mL lower layer fluid into the same centrifuge tube above. Samples were concentrated to dry in a vacuum, dissolve samples with 200 μL isopropanol, and the supernatant was filtered through a 0.22 µm membrane to obtain the prepared samples for Liquid chromatography–mass spectrometry (LC-MS), take 20 µL from each sample to the quality control (QC) samples (these QC samples were used to monitor deviations of the analytical results from these pool mixtures and compare them to the errors caused by the analytical instrument itself). Use the rest of the samples for LC-MS detection.

### 2.5. Lipidomic Analysis by LC/MS

Chromatographic separation was carried using a Thermo ultimate 3000 system (Thermo, Waltham, MA, USA) equipped with an ACQUITY UPLC^®^ BEH C18 (100 × 2.1 mm, 1.7 µm, Waters) column (Agilent, Santa Cruz, CA, USA) maintained at 50 °C. The temperature of the autosampler was set on 8 °C; gradient elution of analytes was carried out with acetonitrile: water = 60:40 (0.1% formic acid + 10 mM ammonium formate) (A) and isopropanol: acetonitrile = 90:10 (0.1% formic acid + 10 mM ammonium formate) (B) at a flow rate of 0.25 mL/min. Injection of 2 μL of each sample was performed after equilibration. An increasing linear gradient of solvent A (*v/v*) was used as follows: 0~5 min, 70~57% A; 5~5.1 min, 57~50% A; 5.1~14 min, 50~30% A; 14~14.1 min, 30% A; 14.1~21 min, 30~1% A; 21~24 min, 1% A; 24~24.1 min, 1~70% A; 24.1~28 min, 70% A. 

The ESI-MSn experiments were executed on the Thermo Q Exactive Focus mass spectrometer (Thermo, Waltham, MA, USA) with the spray voltage of 3.5 kV and −2.5 kV in positive and negative modes, respectively; sheath gas and auxiliary gas were set at 30 and 10 units respectively and finally, the capillary temperature was set to 325 °C. The orbitrap analyzer (Thermo Fisher Scientific, Waltham, MA, USA) scanned over a mass range of m/z 150–2000 for a full scan at a mass resolution of 35,000. Data-dependent acquisition (DDA) MS/MS experiments were performed with an HCD scan. The normalized collision energy was 30 eV. Dynamic exclusion was implemented to remove some unnecessary information in MS spectra [23]. Lipid identification and quantification were assessed with Thermo Scientific™ LipidSearch™ (Santa Cruz, CA, USA) developed joinlty by Professor Ryo Taguchi and Mitsui Knowledge Industry Co. (Tokyo, Japan). Lastly, the AUCs (areas under the curve) were normalized to intensity in a lipid class. 

### 2.6. Differential Expression Analysis

Total RNA was obtained using TRIzol method (Invitrogen, Waltham, MA, USA) according to the manufacturer’s protocol with slight modifications. Paired-end sequencing with a read length of 150 bp was performed using an Illumina NovaSeq 6000 platform (Illumina, San Diego, CA, USA) with three biological replicates for each developmental stage. Subsequently, raw read quality evaluation using FastQC (http://www.bioinformatics.babraham.ac.uk/project/fastqc, accessed on 1 January 2020) with default options and adapter sequence trimming using CutAdapt (http://ppypi.python.org/pypi/cutadapt, accessed on 1 January 2020) was conducted to obtain high quality reads. For assembly, Trinity package (http://trinityrnaseq.github.io, accessed on 1 January 2020) was used to assemble the tigernut transcriptome. Assembled contigs were clustered based on shared reads of transcripts by using Corset (https://code.google.com/p/corset-project/, accessed on 1 January 2020), and representative sequences isolated from each cluster were defined as unigenes. Distinct sequences were mapped against various protein and nucleotide databases such as Nr, Nt, Pfam, COG, Swiss-Prot to identify the functional annotation of unigenes. Gene ontology functional classification in the GO was obtained using WeGo (http://wego.genomics.org.cn, accessed on 1 January 2020). FPKM (Fragments Per Kilobase of transcript sequence per Millions base pairs sequenced) method was used for the caculation of transcript expression levels [24]. False Discovery Rate (FDR) control is used to correct for *p*-value in multiple tests using DEseq2 [25]. Differentially expressed genes (DEGs) were identified with |log2 (Fold Change)| > 1 and FDR < 0.05.

### 2.7. Cloning and Functional Identification of CePAH1 Gene

Total RNA was extracted from the tuber tissues of *Cyperus esculentus* using Trizol reagent (Thermo Fisher Scientific, Santa Cruz, CA, USA). RNA concentration was determined with a NanoDrop2000 spectrophotometer (Thermo Scientific, Santa Cruz, CA, USA). Approximately, 1 µg of total RNA was used for the first-strand cDNA synthesis using the PrimeScript™ 1st strand cDNA synthesis Kit (Takara Biomedical Technology Corporation, Beijing, China). The *CePAH1* gene sequence was extracted from the transcriptome data, and primers were designed for amplification. The PCR amplicons were purified from agarose gel (1%) and then cloned into pESC-URA vector for sequencing. For recombinant vector (pESC-URA-*CePAH1*) construction, the full length sequence of *CePAH1* digested with *Not* I and *Cal* I restriction enzymes, and ligation was performed after purification of the digested fragments. The resulting plasmid encodes a fusion protein consisting of *CePAH1* and a C-terminal FLAG tag. For protein expression in *Saccharomyces cerevisiae*, the induced expression of recombinant protein was carried out using 2% galactose, and the induction time was kept in the range of 12 h, 24 h, 36 h, 48 h, 60 h, 72 h and 84 h, respectively. Using yeast total protein extraction kit, the crude protein extraction was performed by collecting the supernatant. Samples were separated on 12% SDS-PAGE and the gel was stained with Coomassie Brilliant Blue G-250, and de-stained gel (with solution of acetic acid:ethanol:H_2_O = 1:3:6) was captured. Finally, the verification of the recombinant *CePAH1* protein expression after induction was carried out using rabbit anti-FLAG Tag antibody (Bioss Biotechnology Company, Beijing, China) by Western blot analysis.

### 2.8. Measurement of DAG and Total Oil Content

A total of 10 g yeast culture sediment was collected from which 2 g sediment was resuspended in PBS buffer solution. After repeated freezing and thawing, the supernatant was collected by centrifugation at 3000 rpm for 20 min, and the DAG content was determined using the Enzyme-linked immunosorbent assay (ELISA) detection method. The remaining 8 g sediment was resuspended in 8 mL deionized water, and centrifuged for 10 min at 13,000 rpm to remove the deionized water. The remaining cells were allowed to dry to a constant weight and then add 40 mL 4 mol/L HCL, followed by a thorough mixing, and then maintained at room temperature for 30 min. After that, boiling for 3 min were performed, and then immediately placed in the refrigerator at −20 °C for 10 min. In the next step, a total volume of 80 mL chloroform: methanol (1:1) was added followed by shaking and proper mixing, and then centrifuged for 25 min at 4000 rpm. About 30 mL of the lower chloroform solution was collected and 30 mL of 15% NaCl solution was added. After mixing, the lower chloroform solution was collected with repeated centrifugation and then allowed to volatilize at room temperature for 36 h. Finally, weigh, and calculate the oil content using the weight difference.

### 2.9. Data Analysis 

Data were subjected to a one-way analysis of variance (ANOVA) to determine the statistical significance. SPSS (version 24) was used for Pearson’s correlation analysis. Differences among different groups were considered statistically significant at *p* < 0.05.

## 3. Results

### 3.1. Raw Oil Contents in Developing Tiger Nut Tubers

**Figure 1:**
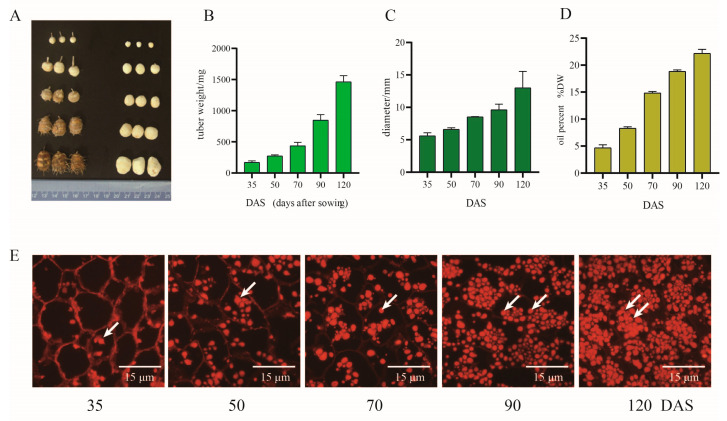
Changes in lipid content and lipid droplets accumulation during tuber development in *C. esculentus*. (**A**) Changes in appearance and growth status of *C. esculentus* tubers. (**B**) Average weight changers per tuber under different development stages. (**C**) Average diameter changes per tuber. (**D**) Draw oil content in dry tubers. (**E**) Lipid droplets accumulation inside the *C. esculentus* tubers. Data indicate means ± SD (*n* = 3).

To investigate the lipid accumulation during different plant tuber developing stages, we analyzed the oil content of different development stages of *C. esculentus*. Tiger nut tubers started to sprout after approximately 3–6 days upon seed sowing under regular growth conditions. The growth period of tubers took around 120 days, while their appearances turned from white to brown; the white appearance was observed during the early stage (35–50 DAS, days after sowing), turned into light brown during the middle stage (50–70 DAS), moderate brown during the late stage (70–90 DAS), and finally turned to dark brown during the mature stage (90–120 DAS). The inside color of the tuber is always oyster white when the coat is removed (Figure 1A). During tuber development, the fresh weight and diameters of each tuber increased significantly following averages of 0.2 g and 0.2 mm per day, respectively (Figure 1B,C). We also determined the oil content during tuber development which showed that raw oil content in tubers increased continuously from 4.6% to 22.2% throughout all developmental stages (Figure 1D). There was a rapid increase in oil accumulation for the early and middle stages, which increased approximately on average 0.26% d^−1^ from 35 to 50 DAS and 0.33% d^−1^ from 50 to 70 DAS, respectively. That fascinating increase was followed by the late and mature stages, which increased approximately on average 0.25% d^−1^ and 0.11% d^−1^ during the late and mature stages, respectively.

The reserves of lipid droplets represent the main storage organelles for natural lipids in plants [26]. To evaluate the variation of lipid accumulation during the developmental process inside the tuber, we stained tubers of different stages with Nile Red to observe the structure and amount change of lipid droplets (LDs) (Figure 1E). The LDs showed irregular spherical structure and a constantly increasing amount with tuber development. The accumulation of lipids in tubers was visually evident; this may indicate that the increase in the expression level of the TAG leads to an increase in the crude oil content, which classifies TAG as a major contributor to the enhancement of oil accumulation in oil plants. 

### 3.2. Lipid Composition and Variation 

**Figure 2:**
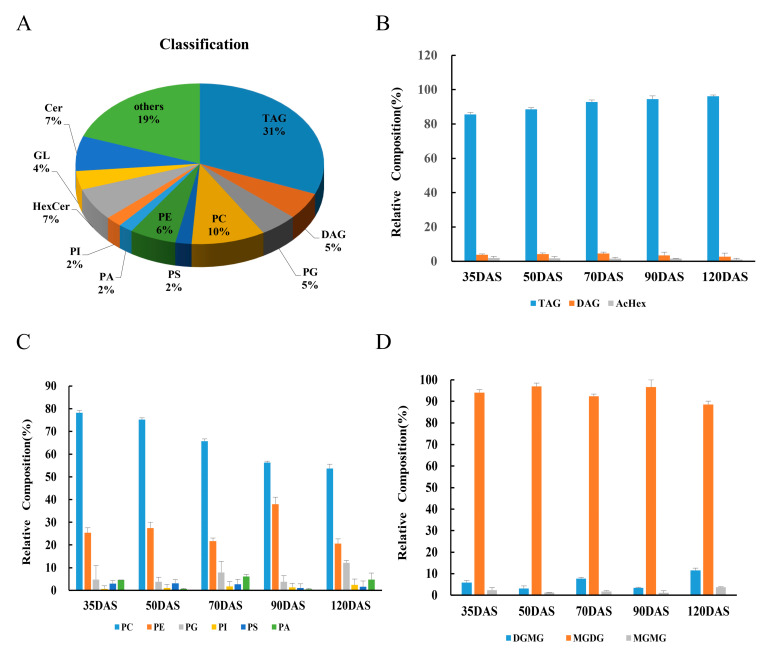
Lipids classification and composition during tuber development. (**A**) Lipids classification and composition; **(B**). Relative proportion of Triacylglycerol composition. (**C**). Relative proportion of phospholipid composition. (**D**). Relative proportion of glycolipids composition. Five individual biological samples are demonstrated, each of which was analyzed with technical six duplicates (means ± SD). Five developmental stages were analyzed.

To determine the changes in the overall lipid composition and distribution in *C. esculentus* tubers, we isolated metabolites from tubers and explored them by LC-MS analysis. Over 430 different lipid types in tubers, consisting of 133 triacylglycerols (TAG) types (31.1%), 23 diacylglycerols (DAG) types (5.4%), 21 phosphatidylglycerols (PLs) types (4.9%), and other lipid types (Figure 2A). As the main neutral lipid’s component found in tubers, TAG content continuously increased until the fully mature stage (Figure 2B). It corroborated that the increased content of lipids is mainly justified by the continuous accumulation of TAG. Besides glycerides, the presence of glycolipids and phospholipids are the main lipids classes in the development of tiger nut tubers. During the phospholipid profiling analysis, the relative composition of PC was continuously decreased along the five different stages of tubers whereas the profiling of PE displayed a minor decrease at the five stages and only a periodical increase at the late stage (Figure 2C). The decrease of PC, periodic decreased of PE and relative content increased of TAG suggests that during the development of tubers, PC is probably converted into TAG and PE might be converted into TAG only in the early and middle stages. Importantly, the glycolipids profile changes lacked regularity, and the metabolism correlation with glycerides was weak (Figure 2D).

### 3.3. Changes in Lipids Accumulation during Tuber Development 

**Figure 3:**
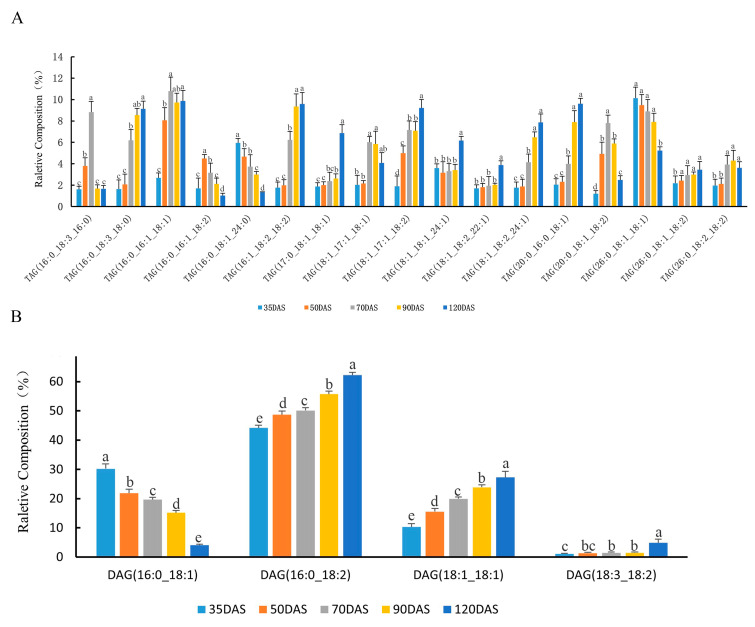
Profiling of different molecular types of glycerolipid classes during tuber development. Triacylglycerol (**A**) and Diacylglycerol (**B**) molecular types were analyzed by LC/MS as described in Materials and methods. Five developmental time points were analyzed, 35, 50, 70, 90, and 120 days after sowing (DAS) for oil accumulation. One-way analysis of variance (ANOVA) was carried out to compare statistical differences (Ducan, *p* <  0.05), Data are means ± SD (*n* = 6).

Rapid lipid accumulation was observed in both the early and middle stages of tuber development. Then, we analyzed the changes occurred in the accumulation of various molecular types of lipids during different stages in tubers. The most prominent type of TAG at all developing stages was 16:0/16:1/18:1, with average relative content higher than 8% at all stages. Other relative abundant types were 16:1/18:2/18:2, 18:1/17:1/18:2, 18:1/18:2/24:1, 16:0/18:3/18:0, and 20:0/16:0/18:1 which contained at least one unsaturated fatty acyl and showed significant increasing tendency in developing stages (Figure 3A). It showed a general shift towards the rising proportion of unsaturated TAG molecular types, leading to an increase of unsaturated fatty acid content in the lipids during the oil accumulation period. Two TAG molecular types with a regular decrease in relative proportion both contained a single 16:0 fatty acyl (16:0/16:1/18:2, 16:0/18:1/24:0). The relative content of molecular types more than 1% at all stages were described here, and changes in the percentages of other low content TAG molecular types were shown in Supplementary Table S1. These variations observed in the formation of TAG molecules partly define the overall lipid composition of *C. esculentus* tubers.

Major molecular types of DAG are displayed in Figure 3B. The most abundant types in DAG at all stages of oil accumulation were 16:0/18:2 and 18:1/18:1, both of these two types increase in abundance during the developmental stages. The relative proportion of molecular types of 16:0/18:1 strongly decreased during lipid accumulation stages whereas molecular types of 18:3/18:2 only increased in the last stage. These significant molecular types of DAG were prevalent with fatty acyl including palmitoyl, oleoyl, or linolenyl. Other less abundant DAG molecular types were shown in Supplementary Table S2.

**Figure 4:**
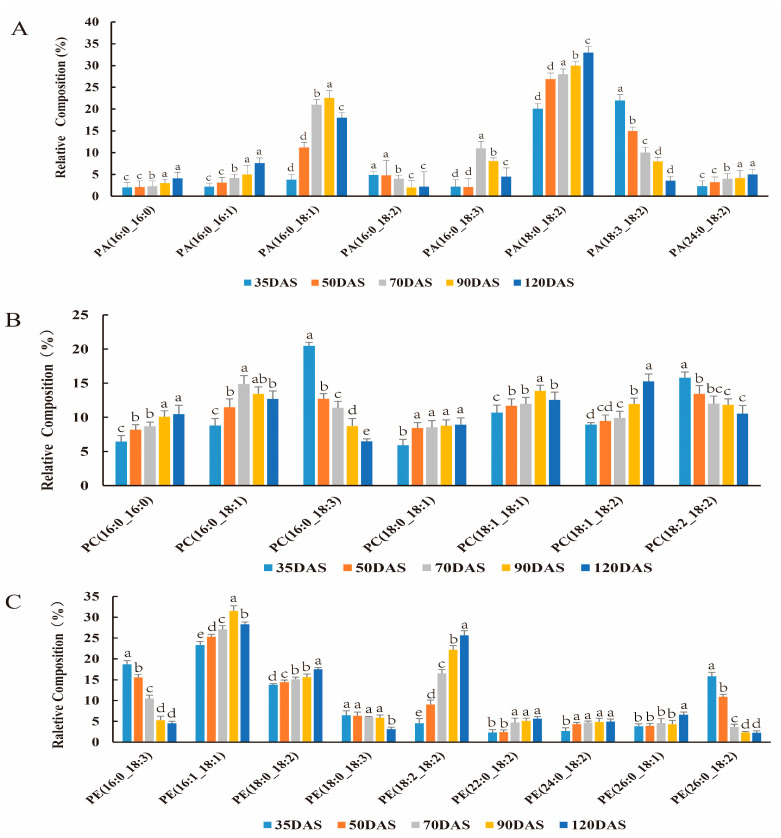
Profiling of different molecular types of phospholipids during tuber development. Phosphatidic (**A**), phosphatidylcholine (**B**), and phosphatidylethanolamine (**C**) molecular types were separated by LC/MS as detailed in Materials and methods. Data indicate means ± SD (*n* = 6).

As immediate precursors of DAG in the Kennedy pathway, the main molecular types of PA are well displayed in Figure 4A. The major types of PA were 16:0/18:1, 18:0/18:2, and 18:3/18:2. During 35 and 120 DAS, the abundance of 18:0/18:2 types significantly increased, while 18:3/18:2 undergo a constant decrease. The 16:0/18:1 types kept the increase in the previous four-time points and slightly decreased in the last time points. Other significant PA types were 16:0/16:0, 16:0/16:1,16:0/18:3,16:0/18:2 and 24:0/18:2. In general, proportions of accumulation of the major PA types constantly increased during the whole process. 

In oil crops, almost all fatty acyl contributed in TAG flux through PC [27], hence PC played a very important role during oil accumulation. The main molecular types of PC during oil accumulation in *C. esculentus* tubers are shown in Figure 4B, while a complete analysis of all detected types is described in Supplementary Table S3. The main molecular types at 35 DAS were 16:0/18:3 and 18:2/18:2, however, both types showed a significantly decreased trend in all development stages. Molecular types 16:0/16:0 and 18:1/18:2 showed an increasing trend during the developmental process. Some other molecules also presented a discontinuous trend of proportion such as, molecular types 16:0/18:1 and 18:1/18:1 was increased between 35 and 70 DAS and slightly decreased in proportion between 90 and 120 DAS. 

The DAG from the Kennedy pathway serves as a substrate for the synthesis of PC as well as PE, the two phospholipids have a strong correlation in the metabolism [28]. Therefore, we examined PE molecular types to compare with those of PC. The main molecular types of PE are shown in Figure 4C with a complete breakdown of all types detected in Supplementary Table S4. The pattern of PE molecular types is similar to that of PC, which is 16:0/18:3 and 18:2/18:2, showed significantly decreased tendency in proportion according to the plant development. In Figure 4C, the major types of PE that showed the most significant pattern were measured 16:1/18:1 and 18:2/18:2, which increased in proportion. In general, other molecular types of PE maintained relatively steady proportions during oil accumulation. These results demonstrate that the PE is synthesized through the same pathway as the PC, but have differential distribution in molecular types proportions.

### 3.4. Transcriptome Analysis of C. esculentus during Tuber Development

Studies on the molecular mechanism of lipid biosynthesis during the development of tiger nut are limited and the genetic resource of the tiger nut plant is still insufficient. In this study, the expression analysis of relevant metabolic genes correlated with lipid metabolism (*PAH1*, *PLD*, *FATA*, *SAD*, *DGAT*, etc.) was carried out by Illumina RNA-seq approach. More than 744 million clean reads (Description of data showed in Supplementary Table S5) and 150,153 unigenes were generated from five libraries. A total number of 8, 2008, 3967, and 7345 different expressed genes corresponding to 50 vs. 35, 70 vs. 35, 90 vs. 35, and 120 vs. 35 DAS were identified, respectively (overall comparison results showed in Supplementary Figure S1). When comparing gene expressions across the five stages of tiger nut tuber’s development, it appeared that the proportion of the expressed genes during the developing stage simultaneously increase along with the plant growth. 

**Figure 5:**
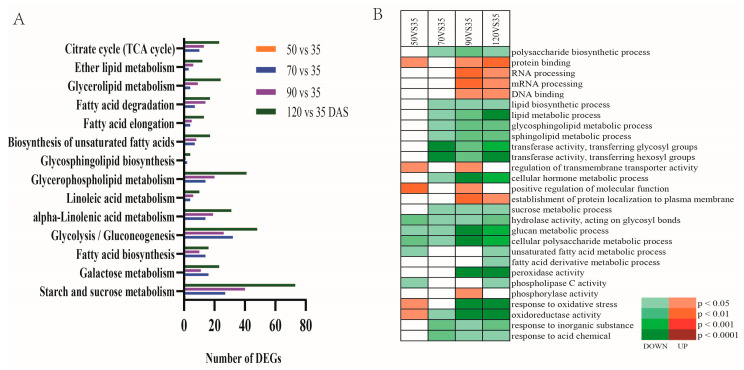
Function annotation of unigenes during tuber development. (**A**) Functional enrichment analyses using the Kyoto Encyclopedia of Genes and Genomes (KEGG) pathways. (**B**) Functional enrichment analyses using GO (Gene Ontology) analysis. Heatmap statistics showing significantly (*p* < 0.05) enriched Gene Ontology categories among the differential expression genes of different development stages. Color gradients toward red indicate high statistic significant GO of up-regulated DEG, Color gradients toward green indicate high statistic significant GO of down-regulated DEG, and blank indicates no statistical significance.

Additionally, physiological analyses revealed different gene expression patterns of each developmental stage, which were mapped to the pathways in the biological pathways database of Kyoto Encyclopedia of Genes and Genomes (KEGG). These differentially expressed genes participated in various lipid metabolic-related processes including linoleic acid metabolism, glycerophospholipid metabolism, etc. (Figure 5A). In addition, all the expressed genes involved in carbohydrate metabolic pathways such as starch and sucrose and glycolysis were found in abundance during the late-developing stages. These findings suggested that G6P and Acyl-CoA may serve as a precursor for the synthesis of lipid in the late-developing stage.

The GO enrichment analysis of differentially expressed genes in tiger nut identified several biological processes, which probably represented specific or common conserved functions of those expressed genes throughout the plant development (Figure 5B). Various functions, such as RNA processing, DNA binding, and protein binding were significantly enriched in the genes with higher expression patterns, particularly at the 90th and 120th DAS developing stage. Likewise, GO terms consisting; responses to oxidative stress, oxidoreductase activity, and peroxidase activity were also significantly enriched for down-regulated genes at 90 and 120 DAS developing stages when compared with 35 DAS. In contrast, these terms were also significantly enriched for two gene sets exhibiting up-regulated expression pattern at 50 vs. 35 DAS stages. Gene sets enriched on the metabolism process of macromolecular compounds like lipid metabolic process, lipid metabolic process, glycosphingolipid metabolic process, sphingolipid metabolic process, exhibited down-regulated expression pattern at 70, 90, and 120 when compared with 35 DAS stage. On the contrary, polysaccharide biosynthetic process indicated higher activity of lipids biosynthetic at initial developing stage. Interestingly, during 120 vs. 35 DAS stages, we observed significant enrichment of GO term in fatty acid metabolism, including the fatty acid derivatives from the metabolic process and unsaturated fatty acid metabolism. Here these genes exhibited down-regulated patterns of tuber development. In addition, genes enriched on transferase activity and phospholipase C activity seemed to be less active at 120 DAS when compared with 35 DAS.

### 3.5. Majors Genes Are Responsible for the Molecular Types Composition of TAG

**Figure 6:**
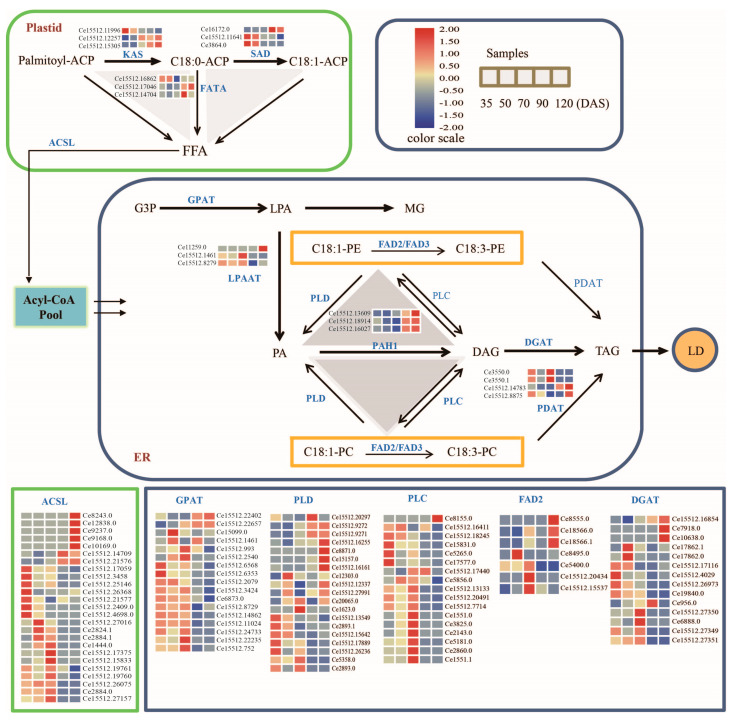
Summary of gene expression profiling in TAG biosynthesis pathway [30] during tiger nut development. KAS, β-ketoacyl-ACP synthetase; SAD, stromal stearoyl-ACP desaturase; FATA, acyl-ACP thioesterase; FFA, Free fatty acid; ACSL, long-chain acyl-CoA synthetase; G3P, glycerol-3-phosphate; GPAT, glycerol-3-phosphate acyltransferase; LPA, lysophosphatidic acid; MGDG, monogalactosyl diacylglycerol; LPAAT, Lysophosphatidic acid acyltransferase; PA, phosphatidic acid; *FAD2/3*, Fatty acid desaturase; PDAT, Phospholipid:diacylglycerol acyltransferase; PLC, phospholipase C; PLD, phospholipase D; PAH1,Phosphatidate phosphohydrolase; DAG, diglycerides; DGAT, diglyceride acyltransferase; TAG, triglycerides; LD, Lipid drop. Heatmap shows the hierarchical clustering of average FPKM values obtained from individual normalized FPKM values of three replicates in five development stages, and colored according to scale bar.

Plant oil quality is usually determined by oil content and fatty acid composition. The unsaturated fatty acyl group composition of TAG is an important factor in evaluating the oil quality. Previously, a total of 12 candidate genes were identified during fatty acid metabolism, elongation in the plastid, lipid desaturation, and synthesis mechanisms of endoplasmic reticulum (ER) through the lipid metabolic pathway of maize [29]. Among these candidate genes, most unigenes of *GPAT1*, *DGAT*, *PLC*, *LPAAT*, *FATA*, and *SAD* showed an abundant transcriptional profile in the early stage and showed dynamic decreased of expression patterns during late and mature stages (Figure 6). Most transcripts of *ACSL*, *PAH1*, *PLD*, and *KAS* have low abundance expression patterns during the initial, early and middle stage and up-regulated expression patterns during the late and mature stage. Two of the *PDAT* unigenes (Ce3550.0, Ce3550.1) were mainly expressed at a high level in 70 DAS; the other two (Ce15512.14783, Ce15512.8875) demonstrated higher expression in the 120 DAS.

The comparative lipidomic analysis also revealed that the unsaturated linolenic acyl group (C18:3) carried by PC, and PE had a more significant decreased level in the mature stage compared with the initial stage, exhibiting 6.76% to 1.45% for PC and 3.85% to 2.93% for PE, respectively. The composition rate of the oleic acyl group (C18:1) decreased by approximately 10.87% in PC and 11.47% in PE during the mature stage when compared with the initial stage (Supplementary Figure S2). Higher transcriptional levels of *PDAT*, *PLC*, and *DGAT* activity could arise from the reduced levels of monounsaturated and polyunsaturated phosphatidylcholine or phosphatidylethanolamines, which are substrates for the Flavin adenine dinucleotide *FAD2* desaturase. *FAD2* desaturase is mainly responsible for the production of polyunsaturated fatty acyl groups in phospholipid. Furthermore, *FAD2* indicated low transcriptional abundance during developing stages, except for the middle stage. The different expression patterns of *FAD2* in ER during developmental stages could explain a portion of the downward trend of oleoyl composition for PC and PE, respectively. Also, the flow of polyunsaturated acyl in PC or PE would partially influence the dynamic variation in the composition of molecular types in TAG.

**Figure 7:**
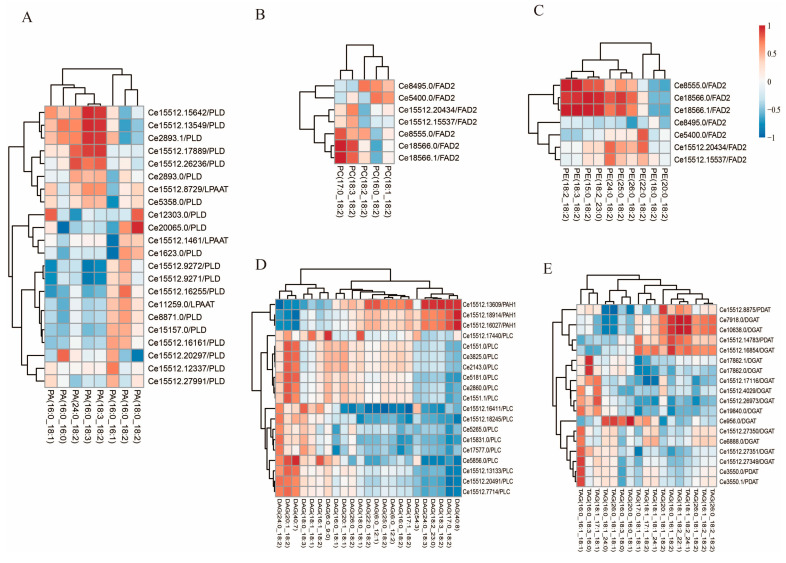
Heat map of the Pearson’s correlation coefficient between gene expression level and intensity of lipid types. The coefficient data is represented by the intensity of the blue or red color as the box at the top right. (**A**) Heat map of the correlation between PA intensity and gene expression levels. (**B**) Heat map of the correlation between PC intensity and gene expression levels. (**C**) Heat map of the correlation between PE intensity and gene expression levels. (**D**) Heat map of the correlation between DAG intensity and gene expression levels. (**E**) Heat map of the correlation between TAG intensity and gene expression levels. Cell colors indicate correlation coefficients from negative (blue) to positive (red).

To gain a better understanding of the relationship between genes and lipid molecular types, the Pearson correlation test was performed for the intensity of molecular types and the expression pattern of unigenes during the tuber development stage. The correlation between essential genes and catalytic products associated with TAG synthesis was identified. Here, molecular varieties and composition of these lipids (PA, PC, PE, and DAG) have important effects on the synthesis and composition of TAG molecular types. Our results showed that a total of 37 transcripts were significantly correlated with 34 molecular types metabolites that exhibit a Pearson correlation coefficient >0.8 and *p*-value < 0.05, and 21 transcripts negatively correlated with 24 molecular types (Figure 7). Among them, the correlation between unigenes and DAG molecular types, *CePAH1* unigenes involved in DAG biosynthesis through the Kennedy pathway were strongly correlated with 19 DAG molecular types, in which three unigenes were positively correlated with expression patterns (Figure 7D).

### 3.6. Expression and Verification of Recombinant Protein

**Figure 8:**
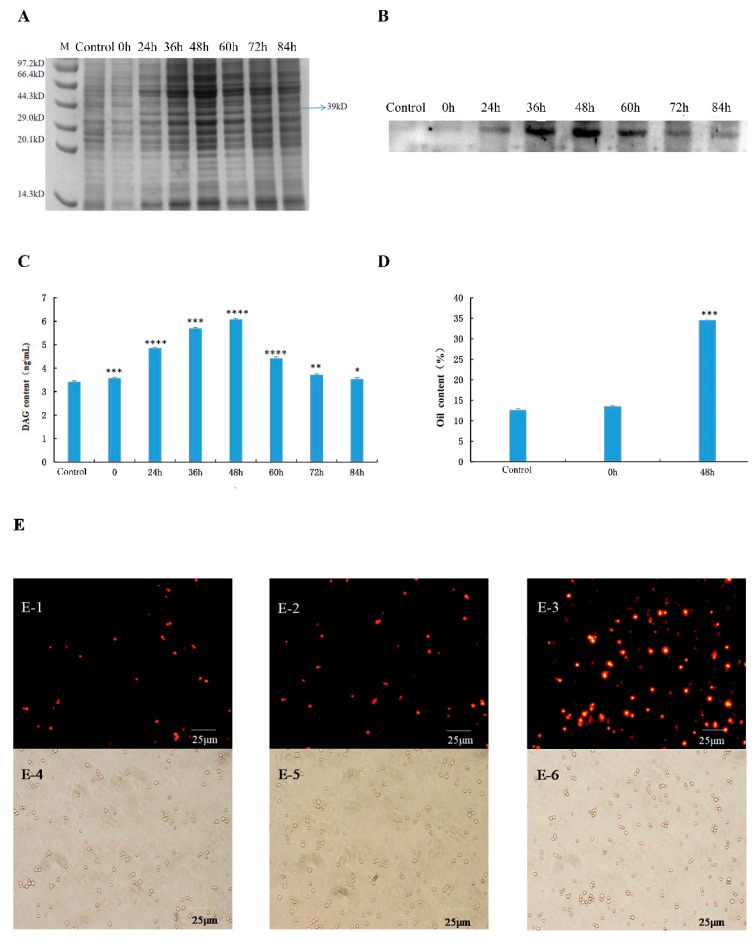
Functional verification of *CePAH1* gene. (**A**) The expression of *CePAH1* protein was detected by SDS-PAGE. (**B**) Western blot analysis of recombination protein at different induction time. (**C**) Content determination of DAG. (**D**) Detection of oil content in yeast cells at different induction time. (**E**) Neil red staining visualization of Yeasts cells under the fluorescence light (**E-1**–**E-3**) and ordinary light (**E-4**–**E-6**). (**E-1**,**E-4)**: Control, Yeast cells with pESC-URA plasmid; (**E-2**,**E-5**): Yeast cells were induced at 0 h; (**E-3**,**E-6**): Yeast cells were induced for 48 h.

The successfully constructed recombinant *Saccharomyces cerevisiae* expression system pESC-URA-*CePAH1* was induced by 2% galactose, and the induction was carried out for 24 h, 36 h, 48 h, 60 h, 72 h and 84 h, respectively. The uninduced pESC-URA vector alone without the target gene was used as control. The target protein of *CePAH1* with a molecular weight of 39 KDa was detected on SDS-PAGE, and the results are shown in Figure 8A, indicating that the recombinant protein was successfully induced.

Western blot results showed the recombinant protein showed a faint line around 39 KDa when it was induced for 24 h compare to the control sample (Figure 8B). The size of the putative *CePAH1* protein was found consistent with the expected size of the recombinant target protein, indicating that the pESC-URA-*CePAH1* engineered bacteria has begun to express the target protein, but the concentration is very low. When induced for 48 h, the target protein line was the most obvious, indicating that the target protein expression began to increase significantly. At 60 h of induction, the target protein expression began to decrease, and at 72 h, the protein expression continued to decrease until 84 h (Figure 8B). The above results indicated that the recombinant *Saccharomyces cerevisiae* expression system pESC-URA-*CePAH1* expressed the target protein when induced by 2% galactose for 48 h, therefore, 48 h was chosen as the optimal induction time for recombinant protein expression.

### 3.7. Detection of DAG Content and Oil Content in Recombinant Saccharomyces cerevisiae Cells

The successfully constructed strain was continuously induced with 2% galactose for 24 h, 36 h, 48 h, 60 h, 72 h, and 84 h to detect the DAG content in *Saccharomyces cerevisiae* cells. The content of DAG in yeast cells is shown in Figure 8C. Consistent with the Western blot results, the highest DAG content in recombinant *Saccharomyces cerevisiae* cells was 6.08 ng/mL at 48 h of induction. Compared with control, the DAG content was increased by 43.75%. The DAG content of 60 h, 72 h, and 84 h of induction, all showed a downward trend, however, compared with 48 h of induction, it was decreased by 27.3%, 38.9%, and 41.9%, respectively. Therefore, it can be seen from the data of DAG content detection that the continuous induction of 2% galactose for 48 h demonstrated the best effect on the synthesis of DAG in the recombinant *Saccharomyces cerevisiae* cells.

The above research shows that 48 h is the best induction time, whereon the DAG content in the recombinant *Saccharomyces cerevisiae* cell showed maximum results. Therefore, we tested the total oil content of the strains on 48 h after induction. As shown in Figure 8D, the highest total oil content in yeast cells was 34.53% at 48 h of induction, and the total oil content in yeast cells without the target gene and uninduced recombinant yeast was 12.67% and 12.75%, respectively. Red staining and observation under a microscope showed that the number of oil droplets in yeast cells induced for 48 h was significantly increased compared with that of recombinant yeast cells that did not contain the target gene and were uninduced (Figure 8E). It proves that *CePAH1* gene can significantly increase oil production in *Saccharomyces cerevisiae* cells.

## 4. Discussion

The utilization of vegetable oil consumption as a source of renewable and environmentally-friendly alternative energy is increasing day by day. The quest for improving crop oil production has been remained a challenging task worldwide. At present, the major source of vegetable oil is traditional seed oil [17]. To enhance the capacity of oil accumulation in plant vegetative tissues such as stems, leaves, and roots is considered one of the effective ways to increase oil production [31,32], which would provide a foundation for developing new sources of plant oils. In this respect, one of the untraditional underground crops, tiger nut (*Cyperus esculentus)* provides a novel and promising platform for plant oil production. The core ability of *Cyperus esculentus* to accumulate oil at high levels (about 25% of dry weight) in the tubers makes them potential target for oil production, which is different from sweet potato and potato that exclusively contain carbohydrates as the major storage component. Tiger nut oil can be used as the rich source of edible oil that contains predominant oleic acid (C18:1) as a source of fatty acid, which is similar to olive oil [33]. As an oil crop with high nutritional value, tiger nut could serve as an ideal plant model to characterize the specific functions and regulation of lipid biosynthesis and storage in plant vegetative tissues. 

A number of pathways involved in lipid metabolism was found to be significant in tiger nut based on the gene function annotation of KEGG and Gene ontology analysis. However, little is known about the dynamic structure of lipid composition and remodeling during tiger nut tuber’s development and maturation. In this study, we have carried out comprehensive lipidomic and transcriptome analyses related to lipid metabolism in different stages of tiger nut tubers development. By using the analysis of dynamic changes in lipid composition and major gene expression profile toward TAG synthesis, the underlying regulatory mechanism of lipid biosynthesis and storage during various developmental phases of tuber development was explored. Our results revealed that the increase of oil content in the tuber during the middle stage (50–70 DAS) was greater than that of tuber oil content during other developing stages. This observation was consistent with the previous study on *C. esculentus,* which showed a rapid lipid accumulation increase in the middle stage (50–85 DAS) [32]. These findings suggested that this stage could be identified as the active stage for lipid biosynthesis and accumulation in tiger nut tubers. 

Neutral lipid represents the highest proportions of lipid in tiger nut. As a major and important lipid stocker [34], the proportion of TAG in tiger nut lipid was shown nearly 87% for the initial stage of tuber development, whereas the proportion of TAG was slightly increased up to 90% for the mature stage. Also, the proportion of PC and PE significantly decreased during plant development and early and middle stage development. Noticeably, it was revealed that PC is the main source for TAG synthesis in plant developing seeds [35], and during the accumulation of TAG, PE does not seems to be as effective as PC [36]. Our results also implied that during the development of plant tubers, PC is actively involved in the synthesis of TAG and PE may actively participate in TAG biosynthesis in the early and middle stages.

Lipids biosynthesis is involved in a series of pathways including fatty acids synthesis (PUSH), lipids biosynthesis (PULL), and (lipids storage) PACKAGE. During PUSH biosynthetic pathways which occurred in plastids, the formation of 18:0-ACP from Palmitoyl-ACP is catalyzed by KAS while the desaturation of 18:0-ACP is catalyzed by stromal stearoyl-ACP desaturase (SAD) to form 18:1-ACP. The acyl-ACP is transferred into free FA through the action of acyl-ACP thioesterase (FATA). Free fatty acids are reactivated by acyl-CoA synthetase (ACSL) and exported from plastids to enter the eukaryotic glycerolipid metabolic pathways [37]. Based on our transcriptome results, abundant *FATA*, *SAD*, and *ACSL* genes guaranteed 16:0, 18:0, and 18:1 free fatty acids synthesized continuously in the plastid, especially in the early stage.

TAG biosynthesis (PULL) in ER is involved in the acylation of glycerol-3-phosphate (G3P) with acyl-CoA and its late dephosphorylation. This phenomenon mainly occurs in the endoplasmic reticulum. During the catalysis of glycerol-3-phosphate acyltransferase (GPAT), lysophosphatidic acid (LPA) was generated from glycerol-3-phosphate (G3P) and transformed into PA through the enzymatic action of LPAAT. Then, DAG was synthesized by the catalytic action of PAH1 and transformed into TAG by the catalytic action of DGAT. DGAT is the rate-limiting enzyme in the Kennedy pathway and plays an important role in the process of TAG synthesis and accumulation. So far, four *DGAT* have been discovered including, *DGAT1*, *DGAT2*, *WS/DGAT*, and soluble *DGAT*. Ectopic expression *of DGAT* gene to improve seed oil content has been previously verified in Arabidopsis [38], soybean [39], corn [40], and other plants. In addition, the role of *DGAT1* and *DGAT2* could be explained in different plants at various development stages, and correlated with the production of TAG with different fatty acid components [41]. For example, in the seeds of castor and tung tree, the expression level of *DGAT2* is higher than that of *DGAT1*, and may plays an important role in the accumulation of oil [42]. Similarly, in tung trees, *DGAT2* showed a strong effect on tung oil acid substrate selectivity [42]. In *Cyperus esculentus* tubers, we also identified two unigenes of *DGAT,* which were up-regulated from 35 DAS to 120 DAS (Ce7918.0 and Ce10638.0) as shown in Figure 6. This may be an effective factor for the accumulation activity of TAG in the tuber development of tiger nut synthesis. 

TAG can also be synthesized by PDAT using PL and DAG as substrates [43]. During the developmental stages, the proportion of C18:1 in PC and PE were decreased drastically in the mature stage. In the same way, the C18:2 and C18:3 fatty acyl types were also declined in the proportion during the PE accumulation course. For the fatty acyl composition of TAG, the 18:1 fatty acid of PE and PC was transported from the plastid to the endoplasmic reticulum, and it was desaturated into C18:2 and C18:3 fatty acid under the catalysis of *FAD2* or *FAD3* [44]. Our RNA-seq results suggested that low abundance of *FAD2* and high expression patterns of PDAT, PLC, and DGAT likely regulate the decent proportion of polyunsaturated fatty acyl in phospholipid, and concurrently affect the molecular types composition of TAG in tiger nut tubers. The synthesis of TAG in ER can be catalyzed by DGAT via the Kennedy pathway or catalyzed by acyl-CoA independent PDAT [35]. Based on the Pearson correlation analysis between unigenes and lipid types, it also implied that biosynthesis of some particular TAG molecular types would be preferred by DGAT or PDAT involved in different pathways, respectively. Furthermore, the molecular composition of TAG types also demonstrated different affects through tuber development. The increased level of PE in the polyunsaturated fatty acyl composition observed in the middle stage is probably related to the high expression of *FAD2* in the middle stage of tuber development. Similar changes were not observed during the PC content analysis, this may be due to the specificity of the enzyme or the phospholipid variety [45]. On the other hand, correlation analysis of gene expression and intensity of lipid molecular types also indicated that the biosynthesis of some lipids was preferred by specific unigenes.

Phosphatidic acid phosphatase (PAH1), a key enzyme that catalyzes the dephosphorylation reaction of phosphatidic acid to produce diacylglycerol (DAG), has become an important regulator of lipid homeostasis in eukaryotes [46]. In the Kennedy pathway, phosphatidic acid (PAs) is used as a precursor to synthesize DAG, which can synthesize phosphatidylcholine (PC) [47]. In addition, PA/DAG also plays a molecular regulatory role in different branches of lipid metabolic pathway such as, lipid signaling, lipid droplet formation, vesicle transport, phospholipid synthesis gene expression, etc. [48,49]. It has been elucidated that PAH1 controls the biosynthesis of TAG and phospholipid membranes and the abundance of lipid signaling molecules [50]. Similarly, PAH1 in the yeast catalyzes the Mg^2+^ dependent dephosphorylation of PA, producing DAG at the nuclear/ER membrane. [51]. Yeast cell mutants lacking this enzyme showed abnormal expansion of the nuclear and endoplasmic reticulum membrane, which was attributed to the increased PA levels and phospholipid synthesis, while down-regulation of TAG biosynthesis [52]. In agreement with the previous results, our findings also revealed that the recombinant protein of *CePAH1* was efficiently induced in yeast cells. Noticeably, the increased DAG and oil content was detected under the catalysis of exogenous *CePAH1*, which preliminarily verified the function of PAH1 in the tiger nut tuber development. In future studies, emphasis on PAH1-induced regulation in plasma membrane and/or cytoplasm, followed by enzymatic dephosphorylation of PA in lipid homeostasis is still required.

## 5. Conclusions

Our study provides a comprehensive overview of lipid composition during different developmental stages of tiger nut tuber. We aimed to use an integrated approach in order to construct the acyl flow of fatty acids involved in the biosynthesis of TAG during tuber development. The transcriptional pattern of the core lipid metabolic pathway genes in *C. esculentus* tubers was also demonstrated and correlations between gene expression pattern and metabolite molecules have also been revealed during tuber developing stages. Overall, the extensive gene mining together with the lipidomic and transcriptome analysis of lipids molecular types allowed the tentative assignment of 37 candidate genes including *CePAH1* during tuber development of tiger nut. The *CePAH1* protein expression and quantitative DAG and TAG analysis of yeast eventually confirmed that *CePAH1* up-regulates DAG content and increased intracellular TAG content. This work paves the way in deeply understanding the underlying molecular mechanism of lipid metabolism during different developmental stages of oil crops.

## Data Availability

Availability of data and materials. The datasets has been uploaded to the NCBI SRA database. SRA accession: PRJNA681247. SRA records will be available with the following link: https://www.ncbi.nlm.nih.gov/sra/ PRJNA681247, accessed on 1 January 2020.

## Supplementary Materials

The following are available online at https://www.mdpi.com/article/10.3390/genes12101492/s1, Figure S1: Overall comparison of different expressed genes under different developing stages of tubers. Figure S2: Comparative analysis of PC. (A) and PE (B) containing unsaturated fatty acyl during tuber developing stages. Table S1: Relative composition of TAG molecular types during five developing stages of tuber. Table S2: Relative composition of DAG molecular species during five developing stages of tuber. Table S3: Relative composition of PC molecular species during five developing stages of tuber. Table S4: Relative composition of PE molecular species during five developing stages of tuber. Table S5: Dataset statistics of transcriptome sequencing of tiger nut tuber during five developing stages.

## Abbreviations

TAG	triacylglycerol
ESI-MS	electrospray ionization mass spectrometry
PC	phosphatidylcholines
PE	Phosphatidyl ethanolamine
DAG	diacylglycerol
LPAAT	lysophosphatidic acid acyltransferase
DGAT	diglyceride acyltransferase
PDAT1	phospholipid:diacylglycerol acyltransferase 1
DAS	days after sowing
OBs	oil body
QC	quality control
SAD	stromal stearoyl-ACP desaturase
FA	fatty acid
LACS	long-chain acyl-CoA synthetase
G3P	glycerol-3-phosphate
GPAT	glycerol-3-phosphate acyltransferase
LPA	lysophosphatidic acid
AcHex	acetylhexosamine
