# Supplementary material for: Tracing Key Molecular Regulators of Lipid Biosynthesis in Tuber Development of Cyperus esculentus Using Transcriptomics and Lipidomics Profiling
Source: Genes (Basel). 2021 Sep 24;12(10):1492. doi: 10.3390/genes12101492 (PMC8535953; doi:10.3390/genes12101492)

Supplementary Figure s1. Overall comparison of different expressed genes under different developing stages of tubers.

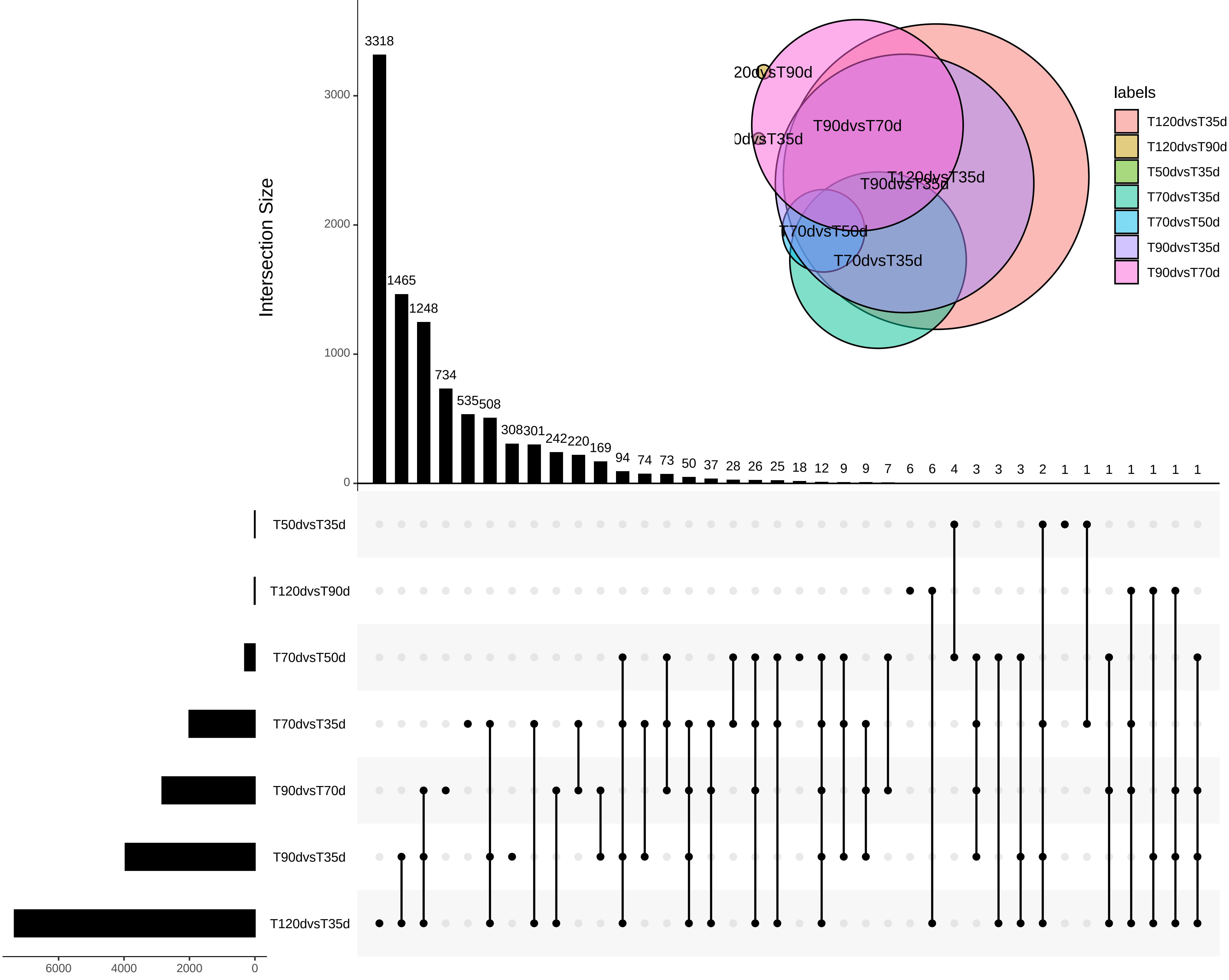

Supplement: Supplementary file 1 [file genes-12-01492-s001.zip › supplementary Figure S1.pdf]
