# Supplementary material for: Tracing Key Molecular Regulators of Lipid Biosynthesis in Tuber Development of Cyperus esculentus Using Transcriptomics and Lipidomics Profiling
Source: Genes (Basel). 2021 Sep 24;12(10):1492. doi: 10.3390/genes12101492 (PMC8535953; doi:10.3390/genes12101492)

Supplementary figure S2: Comparative analysis of PC.
(A) and PE (B) containing unsaturated fatty acyl during tuber developing stages.

A

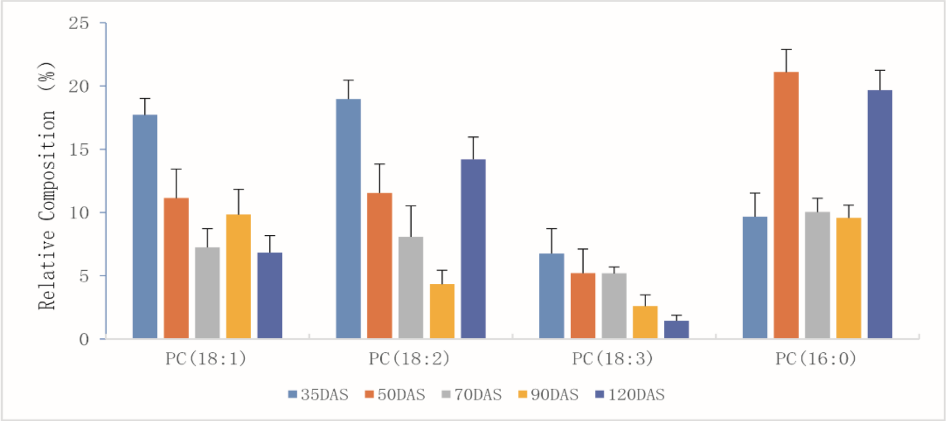

B

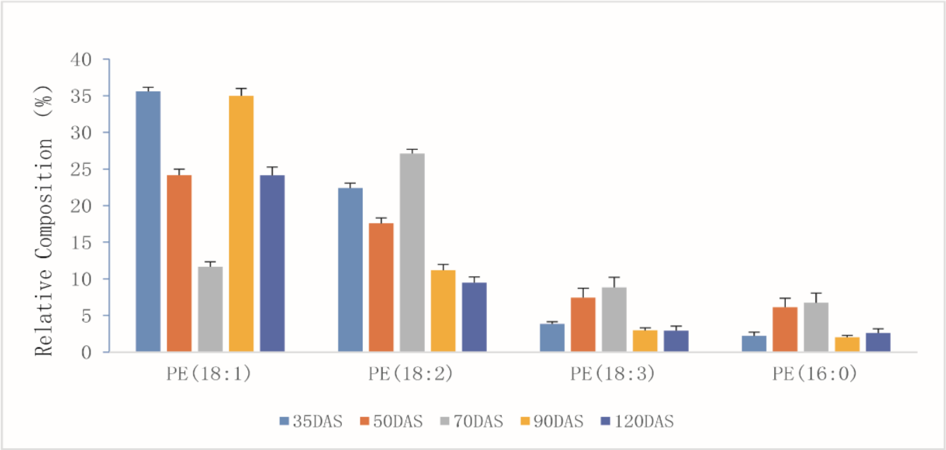

Supplement: Supplementary file 1 [file genes-12-01492-s001.zip › Supplementary Figure S2.pdf]
