# Supplementary material for: Tracing Key Molecular Regulators of Lipid Biosynthesis in Tuber Development of Cyperus esculentus Using Transcriptomics and Lipidomics Profiling
Source: Genes (Basel). 2021 Sep 24;12(10):1492. doi: 10.3390/genes12101492 (PMC8535953; doi:10.3390/genes12101492)
Supplement: Supplementary file 1 [file genes-12-01492-s001.zip › Supplementary table S1.pdf]

Supplementary table S1: Relative composition of TAG molecular types during five developing stages of tuber

	35DAS	50DAS	70DAS	90DAS	120DAS
TAG(16:0_18:3_17:0)	0.007732874	0.008346907	0.00371452	0.005550724	0.002595366
TAG(10:0_18:1_18:2)	0.00147716	0.008939086	0.000125985	0.000492966	0.000323611
TAG(10:0_18:2_18:2)	0.009427293	0.000258532	0.000114312	0.000362119	0.000124317
TAG(12:1e_6:0_18:2)	0.007924751	0.000331363	0.000249001	0.000225341	0.000202425
TAG(12:1e_6:0_18:3)	0.006913365	0.000197531	0.00020841	0.000333573	0.000183596
TAG(14:0_18:2_18:2)	0.008383147	0.009988825	0.003902653	0.009709753	0.00303566
TAG(15:0_16:0_18:1)	0.009346774	0.006913825	0.001684883	0.002594418	0.001000718
TAG(15:0_16:0_18:2)	0.005363653	0.002470694	0.003678287	0.001087193	0.001723628
TAG(15:0_16:1_18:2)	0.009649319	0.002047967	0.000439852	0.000550369	0.000467888
TAG(15:0_18:1_18:2)	0.007310449	0.00941106	0.005175335	0.007715347	0.003841157
TAG(15:0_18:2_18:2)	0.009399486	0.008613968	0.00204756	0.001090246	0.001625932
TAG(15:0_18:2_18:3)	0.001754137	0.001793853	0.001211989	0.001129811	0.000900001
TAG(15:0_18:3_18:3)	0.007186474	0.000539455	0.000842662	0.000420153	0.000504773
TAG(16:0_10:0_10:1)	0.00366291	0.000392049	0.000217063	0.000557084	0.000265054
TAG(16:0_10:0_18:1)	0.009635033	0.005809065	0.00017387	0.000744787	0.000366214
TAG(16:0_10:1_10:1)	0.004129927	0.000130841	0.000146498	0.001473957	8.89263E-05
TAG(16:0_12:0_18:1)	0.005239627	0.000747024	0.000236416	0.000755821	0.000448683
TAG(16:0_12:0_18:2)	0.00935167	0.000816499	0.000468836	0.000703096	0.000545696
TAG(16:0_12:3_14:4)	0.009128174	0.003197488	9.60442E-05	0.000111342	0.00010445
TAG(16:0_14:0_18:1)	0.00581828	0.002189943	0.001220148	0.002852108	0.001077831
TAG(16:0_14:1_18:1)	0.003958124	0.001076369	0.00052504	0.001076881	0.000538676
TAG(16:0_14:2_18:1)	0.002244582	0.00077513	0.000620443	0.000934474	0.000502545
TAG(16:0_16:0_24:0)	0.0097749	0.001174315	0.000824851	0.0007287	0.000185758
TAG(16:0_16:0_24:1)	0.009219123	0.00716483	0.002735633	0.005180737	0.002255182
TAG(16:0_17:0_18:1)	0.004200709	0.002829728	0.000953027	0.002260503	0.001106923
TAG(16:0_17:1_18:1)	0.004014303	0.005016011	0.00269512	0.001073255	0.001698867
TAG(16:0_18:1_18:3)	0.001973443	0.000279023	0.000209135	0.000370819	0.000191336
TAG(16:0_18:1_19:0)	0.005153637	0.008718107	0.003468631	0.002122981	0.000940296
TAG(16:0_18:1_21:0)	0.001545238	0.001307153	0.001570847	0.001561325	0.000849397
TAG(16:0_18:1_23:0)	0.0022221	0.001127071	0.001657782	0.001152387	0.000675234
TAG(16:0_18:2_24:0)	0.001962596	0.001446447	0.001838648	0.000769197	0.001779091
TAG(16:0_18:3_18:3)	0.003642822	0.004542689	0.00337552	0.002318175	0.003662189
TAG(16:0_6:0_12:0)	0.000515578	0.000438898	0.000413407	0.000412674	0.000330032
TAG(16:0_6:0_12:1)	0.004105462	0.000278414	0.000826872	0.000170307	0.000433672
TAG(16:0_8:0_23:1)	0.002774963	0.000279018	0.000462662	0.000238855	0.00022407
TAG(16:1_18:1_18:1)	0.004151824	0.000492786	0.000156327	0.000831752	0.000250196
TAG(16:1_18:1_18:2)	0.004894506	0.005943749	0.004292807	0.004001371	0.002181881
TAG(16:1_18:3_18:3)	0.001548451	0.000887414	0.000676212	0.000331648	0.00031112
TAG(18:0_10:0_10:1)	0.000469827	0.002210944	0.000121158	0.00047376	0.000375258
TAG(18:0_6:0_20:4)	0.000330036	0.000299145	0.000257796	0.000256732	0.000206164
TAG(18:1_10:1_10:1)	0.003553803	0.000501805	0.000242939	0.000844252	0.000375711
TAG(18:1_11:1_12:2)	0.001742427	0.000195457	0.000107193	0.000147391	8.55301E-05

TAG(18:1_18:1_18:3)	0.002702631	0.000438143	0.00017942	0.000267531	0.000204136
TAG(18:1_18:1_21:0)	0.005885505	0.001722992	0.000731858	0.000808065	0.000758975
TAG(18:1_18:1_21:1)	0.00168878	0.001376721	0.000804835	0.001602425	0.000835062
TAG(18:1_18:1_23:0)	0.004244649	0.001344573	0.003213625	0.001702145	0.001078367
TAG(18:1_18:2_22:4)	0.007095605	0.000435168	0.000662982	0.000444552	0.000349469
TAG(18:1_18:2_23:0)	0.006648414	0.001386967	0.000981982	0.001720878	0.001023846
TAG(18:2_10:1_10:1)	0.008623947	0.005567271	0.000194779	0.000446909	0.000233257
TAG(18:2_13:0_18:2)	0.005005169	0.00060099	0.000420674	0.000454757	0.000463557
TAG(18:2_14:1_18:2)	0.005671515	0.002019547	0.000922624	0.001297321	0.000559693
TAG(18:2_14:2_18:2)	0.009106119	0.001291263	9.2007E-05	0.000202784	0.00010006
TAG(18:2_17:1_18:2)	0.001314833	0.003464951	0.002493165	0.001210429	0.000755486
TAG(18:2_18:2_21:0)	0.005564748	0.001673479	0.001160793	0.0015317	0.001184151
TAG(18:2_18:2_23:0)	0.002242465	0.001534706	0.001926874	0.001665307	0.001351408
TAG(18:3_18:2_18:3)	0.002958345	0.003938155	0.004138351	0.000855997	0.001392251
TAG(18:3_18:2_23:0)	0.004122569	0.0004288	0.000626284	0.000367077	0.00040476
TAG(18:4_6:0_16:0)	0.004566678	0.001808315	0.000232504	0.000263388	0.000208753
TAG(19:0_18:1_18:1)	0.009239407	0.007059877	0.001564133	0.003036945	0.001418729
TAG(19:1_18:1_18:1)	0.006402007	0.006980545	0.000986084	0.001265353	0.000758684
TAG(19:1_18:1_18:2)	0.002707722	0.003353952	0.000515552	0.002545027	0.001460596
TAG(19:1_18:2_18:2)	0.002382845	0.001465506	0.00135314	0.002129753	0.00132301
TAG(19:1_18:2_18:4)	0.009098573	0.001671218	0.000163511	0.000515637	0.000152821
TAG(20:1_18:1_18:2)	0.009639444	0.008192689	0.003369518	0.008131076	0.001471342
TAG(20:1_18:2_18:2)	0.00582542	0.005307026	0.006968126	0.003751214	0.001634266
TAG(20:2_18:2_18:2)	0.008462554	0.007189053	0.000204839	0.000623899	0.0001911
TAG(20:3_18:2_18:2)	0.009251725	0.009422104	0.000188625	0.000218668	0.000328598
TAG(22:0_18:2_18:3)	0.008051827	0.001970945	0.002858975	0.000507196	0.000542959
TAG(24:0_18:2_18:3)	0.006138091	0.001044204	0.002080029	0.001023189	0.000402109
TAG(25:0_16:0_16:0)	0.009218492	0.007555047	0.000280785	0.0001898	0.000155411
TAG(25:0_16:0_18:1)	0.003882224	0.005610714	0.002688671	0.002134632	0.001366072
TAG(25:0_18:1_18:1)	0.009169267	0.006436144	0.001437584	0.002228874	0.00117116
TAG(25:0_18:1_18:2)	0.006237055	0.006035269	0.000857763	0.004657045	0.001073182
TAG(25:0_18:2_18:2)	0.008854531	0.004979434	0.001428072	0.002159993	0.002414096
TAG(26:0_16:0_16:0)	0.009028548	0.008347793	0.000302058	0.001508606	0.00075668
TAG(26:0_16:0_18:1)	0.013079063	0.005822774	0.004893442	0.003213608	0.001707823
TAG(26:0_18:0_18:1)	0.008542857	0.00805946	0.002363105	0.003124148	0.00111876
TAG(26:0_18:2_18:3)	0.005395966	0.003337223	0.004555879	0.001137621	0.002018137
TAG(27:0_16:0_18:1)	0.009355308	0.003404114	0.000944956	0.000405959	0.000276526
TAG(27:0_18:0_18:1)	0.009376369	0.012701259	0.000179037	0.00047684	0.00028278
TAG(27:0_18:1_18:1)	0.007553999	0.008673398	0.003297204	0.004166486	0.001886822
TAG(27:0_18:1_18:2)	0.003472736	0.005100523	0.003063699	0.002674093	0.001805026
TAG(27:0_18:2_18:2)	0.009385888	0.004468896	0.00337042	0.002114832	0.001516066
TAG(28:0_18:1_18:1)	0.007785382	0.012587326	0.004023149	0.00331025	0.001355923
TAG(28:0_18:1_18:2)	0.005811236	0.014953981	0.004076217	0.001887252	0.002190589

TAG(28:0_18:2_18:2)	0.008400777	0.00704336	0.001653665	0.001216526	0.000618854
TAG(29:0_18:1_18:2)	0.009189863	0.009391434	0.000169921	8.08722E-05	0.000189322
TAG(30:0_16:0_18:1)	0.009174662	0.010671394	0.000738456	0.000415003	0.000282543
TAG(6:0_12:0_18:1)	0.00811279	0.015065914	0.000321331	0.000379712	0.000324942
TAG(6:0_12:0_18:3)	0.009088504	0.010739015	0.000155152	7.34495E-05	4.31377E-05
TAG(6:0_12:1_18:2)	0.010878023	0.007211263	0.000829445	0.000174555	0.000541087
TAG(6:0_6:0_6:0)	0.009184195	0.015901566	0.000407273	7.2361E-05	0.000243999
TAG(8:0_18:1_18:1)	0.008804984	0.006510249	0.0001914	0.00038241	0.000261898
TAG(8:0_8:0_10:0)	0.008162353	0.006294094	3.22345E-05	0.001315768	4.25621E-05
TAG(9:0_11:1_18:1)	0.004464161	0.004771766	0.000203876	0.002967103	0.000307305
