# Supplementary material for: Tracing Key Molecular Regulators of Lipid Biosynthesis in Tuber Development of Cyperus esculentus Using Transcriptomics and Lipidomics Profiling
Source: Genes (Basel). 2021 Sep 24;12(10):1492. doi: 10.3390/genes12101492 (PMC8535953; doi:10.3390/genes12101492)
Supplement: Supplementary file 1 [file genes-12-01492-s001.zip › Supplementary table S2.pdf]

Supplementary table s2: Relative composition of DAG molecular species during five developing stages of tuber

	35DAS	50DAS	70DAS	90DAS	120DAS
DAG(16:1_18:2)	0.007986177	0.0073403	0.005182268	0.001900879	0.000269686
DAG(17:0_18:2)	0.00883913	0.007722247	0.003961231	0.002901615	0.00101101
DAG(17:1_18:2)	0.008610044	0.008000963	0.00535676	0.003200354	0.001332459
DAG(18:0_18:1)	0.008486487	0.008295294	0.004838694	0.001900692	0.001340316
DAG(18:0_18:3)	0.008464089	0.008226002	0.005623852	0.001586181	0.00051107
DAG(18:2_23:0)	0.008632306	0.007052828	0.005492744	0.002819649	0.000472385
DAG(20:1_18:1)	0.009065319	0.007793153	0.004755636	0.002833876	0.001329256
DAG(20:1_18:2)	0.008949866	0.008123774	0.005617029	0.002615838	0.001522453
DAG(22:0_18:2)	0.008272984	0.008487469	0.004876889	0.001914696	0.000597618
DAG(24:0_18:2)	0.008678135	0.007563337	0.006302619	0.00267054	0.000738168
DAG(24:0_18:3)	0.008560578	0.008125095	0.004753791	0.002635993	0.001410738
DAG(25:0_18:2)	0.008661754	0.007978621	0.006147016	0.002284531	0.001180157
DAG(26:0_18:2)	0.007997229	0.007946657	0.006408639	0.003121843	0.001579218
DAG(6:0_12:1)	0.008305858	0.007872333	0.006259829	0.00207823	0.001088487
DAG(6:0_12:2)	0.008633061	0.007775991	0.005939021	0.001493127	0.000737578
DAG(6:0_9:0)	0.008471706	0.007501614	0.006197295	0.00173627	0.00050555
