# Supplementary material for: Tracing Key Molecular Regulators of Lipid Biosynthesis in Tuber Development of Cyperus esculentus Using Transcriptomics and Lipidomics Profiling
Source: Genes (Basel). 2021 Sep 24;12(10):1492. doi: 10.3390/genes12101492 (PMC8535953; doi:10.3390/genes12101492)
Supplement: Supplementary file 1 [file genes-12-01492-s001.zip › Supplementary table S3.pdf]

Supplementary table S3: Relative composition of PC molecular species during five developing stages of tuber

	35DAS	50DAS	70DAS	90DAS	120DAS
PC(15:0_18:1)	0.008904	0.009439	0.00874	0.007522	0.0084
PC(16:0_18:2)	0.008992	0.008777	0.009031	0.012601	0.00848
PC(17:0_18:2)	0.008746	0.009219	0.009284	0.008046	0.00833
PC(17:0_18:3)	0.009042	0.009043	0.008848	0.007549	0.008556
PC(18:1_14:0)	0.009307	0.009231	0.008661	0.008662	0.008427
PC(18:3_18:2)	0.00884	0.0095	0.007918	0.007425	0.008486
PC(18:3_18:3)	0.009012	0.009304	0.008538	0.007008	0.008556
PC(22:0_11:2)	0.008992	0.009116	0.007796	0.006915	0.008785
PC(26:0_18:1)	0.00898	0.00903	0.008021	0.007915	0.008274
