# Supplementary material for: Tracing Key Molecular Regulators of Lipid Biosynthesis in Tuber Development of Cyperus esculentus Using Transcriptomics and Lipidomics Profiling
Source: Genes (Basel). 2021 Sep 24;12(10):1492. doi: 10.3390/genes12101492 (PMC8535953; doi:10.3390/genes12101492)
Supplement: Supplementary file 1 [file genes-12-01492-s001.zip › Supplementary table S4.pdf]

Supplementary table S4: Relative composition of PE molecular species during five developing stages of tuber

	35DAS	50DAS	70DAS	90DAS	120DAS
PE(15:0_18:2)	0.008371	0.007456	0.007778	0.00274	0.001794
PE(16:0_18:3)	0.008412	0.007959	0.006862	0.003054	0.001913
PE(16:0_20:1)	0.008079	0.007548	0.006936	0.002489	0.001954
PE(18:1_22:0)	0.007992	0.007459	0.007299	0.003256	0.001909
PE(18:1_24:0)	0.008354	0.007292	0.006799	0.002268	0.001588
PE(18:2_23:0)	0.008335	0.00731	0.006909	0.003296	0.001685
PE(18:3_18:2)	0.00828	0.00801	0.006477	0.003058	0.001707
PE(20:0_18:2)	0.00802	0.007765	0.006919	0.002546	0.001772
PE(25:0_18:1)	0.007969	0.007299	0.007214	0.002656	0.001749
PE(25:0_18:2)	0.008378	0.007902	0.006807	0.002971	0.001429
