# Supplementary material for: Tracing Key Molecular Regulators of Lipid Biosynthesis in Tuber Development of Cyperus esculentus Using Transcriptomics and Lipidomics Profiling
Source: Genes (Basel). 2021 Sep 24;12(10):1492. doi: 10.3390/genes12101492 (PMC8535953; doi:10.3390/genes12101492)
Supplement: Supplementary file 1 [file genes-12-01492-s001.zip › supplementary table S5.pdf]

Supplementary table S5: Dataset statistics of transcriptome sequencing of tiger nut tuber during five developing stages

Sample name	Total Reads	Mapping rate
35DAS-1	51648136	39569964(76.61%)
35DAS-2	50040900	38931528(77.80%)
35DAS-3	60458772	47043460(77.81%)
50DAS-1	58561086	46472058(79.36%)
50DAS-2	40214314	30935464(76.93%)
50DAS-3	52606584	40701820(77.37%)
70DAS-1	46273318	36858328(79.65%)
70DAS-2	60981932	47918946(78.58%)
70DAS-3	39815914	31025786(77.92%)
90DAS-1	46748192	37663982(80.57%)
90DAS-2	42709522	34485304(80.74%)
90DAS-3	52464046	41639592(79.37%)
120DAS-1	40909822	32892554(80.40%)
120DAS-2	57417830	46430524(80.86%)
120DAS-3	43729798	34592926(79.11%)
